# The effect of preparation design on the fracture resistance and adaptation of the CEREC ceramic endocrowns

**DOI:** 10.1002/cre2.726

**Published:** 2023-03-22

**Authors:** Samar Jalali, Hamid Jalali, Mohammad Javad Kharazi Fard, Ali Abdolrahmani, Marzieh Alikhasi

**Affiliations:** ^1^ School of Dentistry Tehran University of Medical Sciences Tehran Iran; ^2^ Department of Prosthodontics, School of Dentistry Tehran University of Medical Sciences Tehran Iran; ^3^ Dental Research Center, Dentistry Research Institute Tehran University of Medical Sciences Tehran Iran

**Keywords:** dental marginal adaptation, endodontically treated teeth, internal adaptation, zirconia lithium silicate

## Abstract

**Objective:**

The purpose of this experimental in vitro study was to assess the effect of having one or two intact axial walls on the improvement of the fracture resistance, and marginal and internal adaptation of computer‐aided design/computer‐aided manufacturer fabricated ceramic endocrowns.

**Materials and Methods:**

Thirty‐six endodontically treated mandibular molars were divided into three groups (*n* = 12). Group A, represented teeth that all of their axial walls were reduced till they all had 3 mm height. In group B, the buccal wall had 5 mm height and the others were reduced to 3 mm. Specimens of the group C had 5‐mm‐height buccal and one 5 mm‐height proximal wall, with all other walls of 3 mm height. All teeth were scanned using intraoral scanner, and endocrowns were milled from zirconia lithium silicate ceramics blocks. The marginal and internal discrepancy of restorations were evaluated with the replica technique. Fracture resistance was also measured after cementation and modes of failure were observed. One‐way analysis of variance and Tukey HSD multiple comparisons were used to analyze the data (*α* = .5).

**Results:**

Significant differences were observed within the groups in terms of the axial discrepancy (*p* = .022); group A had significantly higher amounts of axial discrepancy compared to group B (*p* = .001) and group C (*p* = .003). Preservation of the intact axial walls did not result in a statistically significant decrease in marginal (*p* = .21) and pulpal (*p* = .31) discrepancy values. Also, concerning the fracture resistance no significant difference was observed among the groups (*p* = .51).

**Conclusion:**

Preservation of at least one of the axial walls could reduce the amount of the axial discrepancy and, therefore, improves the adaptation of the restoration. However, based on this study, it did not improve fracture resistance.

## 1 INTRODUCTION

Endocrown was introduced in 1995 as a monoblock restoration connecting the core structure with crown restoration (Pissis, 1995). With the enhancement of adhesive dentistry and a shift toward more conservative procedures, endocrowns were suggested as an alteration for post and core treatment (Biacchi & Basting, 2012; Bindl & Mörmann, 1999). Through the recent advancements in technology, computer‐aided design/computer‐aided manufacturing (CAD/CAMs) are being widely used for the fabrication of the endocrowns. Using this technology, the restorations can be made with precise marginal adaptation and excellent esthetic in a lesser amount of time (Lin et al., 2013; Shin et al., 2017). Moreover, CAD/CAM endocrowns display higher fracture resistance comparing to conventional single crowns (Biacchi & Basting, 2012). When using digital dentistry, several factors should be considered. The features of CAD/CAM‐fabricated restorations can be determined by the scanner type (Hack & Patzelt, 2015), milling strategy (type of the burs) (Zimmermann et al., 2018), and software version (Shim et al., 2015).

Molars specifically the ones with clinically short crowns and narrow or calcified root canals are good candidates for this type of restoration (Dogui et al., 2018). Endocrowns have shown to be a promising restoration for premolars with the same rates of longevity as molars (Thomas et al., 2020). However, due to the lack of data, there is a question about the clinical indication of endocrown for the anterior teeth (Govare & Contrepois, 2020).

Marginal integrity is an important factor in the long‐term success of restorations. An increase in the marginal discrepancy would result in plaque accumulation and dental caries. The other disadvantage of marginal discrepancy is the possibility of cement dissolution (Contrepois et al., 2013). Despite some controversial results, decreasing cavity depth (Rocca et al., 2018), using digital technique (Ghoul & Salameh, 2020; Saglam, Cengiz, & Karacaer, 2020), and meticulous adjustment of the restorations may enhance marginal integrity of endocrowns (M Hasanzade et al., 2019).

The other key factor for endocrowns success is its resistance to fracture. A recent systematic review showed that endocrowns could have similar or even higher fracture resistance compared to conventional crowns (Al‐Dabbagh, 2021). Inadequate thickness of restoration (Turkistani et al., 2019), extra extension into the pulp chamber (Dartora et al., 2018), and type of finishing line are among the factors that can affect fracture resistance of endocrowns (Taha et al., 2018b).

Biomimetic dentistry is a field of science that focuses on the return of the tooth to its function, esthetic, and strength by mimicking the biochemical process and producing the biological materials. One of the fundamental aspects of restorative biomimetic dentistry is minimal invasiveness. That is to say, conserving the maximum healthy dental tissue while removing the defects and caries (Bazos & Magne, 2011; Goswami, 2018; Malterud, 2013; Zafar et al., 2020). According to this concept, while preservation of the tooth structure and remaining of the intact axial walls is a principle of preparation, it can make the preparation design more complicated. Due to the limited accuracy of intra‐oral scanners, adaptability of CAD/CAM restorations becomes more challenging as preparation becomes more complex (de Andrade et al., 2022). Therefore, the purpose of this study was to determine the effect of presence of intact axial walls on the fracture resistance, and adaptation of CAD/CAM endocrowns. The hypothesis tested in this study was that keeping the intact axial walls could improve the restoration's adaptation and its fracture resistance.

## 2 MATERIALS AND METHODS

### 2.1 Samples

Thirty‐six extracted human permanent mandibular and maxillary molars with almost the same bucco‐lingual and mesio‐distal size were selected for this study. The exclusion criteria were carious lesions on the axial walls, very large or very small clinical crown, crown or root fracture, and decalcification on the tooth surface. The Bucco‐lingual and mesio‐distal dimension of the teeth were measured using a digital caliper. Teeth were sorted according to the anatomical shape of their crown and were divided into three groups (*n* = 12) using the stratified randomized allocation technique. Root surfaces were cleaned from soft tissue and calculus using ultrasonic instrument. Teeth were restored in 5% chloramine solution. All endodontic procedures were performed by the same person with the same technique and then, teeth were stored in normal saline solution. Roots of the teeth were coated with a thin layer (0.4 mm) of wax (Azarmoom, Azarshahr, Iran), resembling the periodontal ligament (Alzahrani & Richards, 2018). All teeth were mounted in a vertical position in metallic molds filled with self‐cure acrylic resin (Acropars, Marlik, Iran) and roots were embedded in resin up to the Cemento‐enamel junction.

### 2.2 Teeth preparation

**Figure 1:**
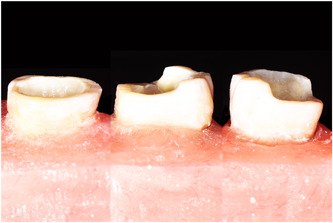
Representative specimens of each group. A: teeth with no complete axial wall. B: teeth with buccal wall remained. C: teeth with buccal and mesial wall remained.

The same cavity preparation was done for all specimens, limited to removing cavity undercuts and to 8°–10° internal tapering (Ghodsi et al., 2021). Butt‐joint finishing line with a width of 1.5 mm was considered as the preparation design for all the teeth. All specimens had their axial walls reduced to 5 mm height from the CEJ. To ensure that all teeth have the same cavity depth extension to the CEJ, we used a flowable composite resin (DX.flow, Dentex) in the pulp chambers of the teeth where the chamber's floor was lower than the CEJ. The irregularities and orifices of the root canals were also sealed and smoothed with a thin layer of flowable composite resin (Hayes et al., 2017). Group A (*n* = 12) represented teeth in which the buccal, lingual, and proximal walls were reduced until they all had 3 mm height. In group B (*n* = 12), the buccal wall, and in group C, the buccal and one of the proximal walls preserved their 5 mm height while the other ones were reduced to 3 mm (Figure 1). The reduction from 5 to 3 mm in all the groups were achieved by drilling 2 mm deep grooves as depth guides. For all the specimens, we used a diamond‐coated stainless steel bur (806314156012, Jota), and after every four preparation we replaced the bur with a new one (Emir et al., 2018).

### 2.3 Endocrown manufacturing

Endocrowns were made using the CEREC AC system (Dentsply Sirona). Teeth were scanned with omnicam (Dentsply Sirona), and CEREC 3D software (version 4.4) was used for designing the endocrowns. To ensure that all fabricated endocrowns were identical, using the biogeneric mode design, the original anatomy produced by the software was used. For all restorations, at least 2 mm occlusal thickness at the reduced walls were considered (Zhang et al., 2022) along with a 60 mm cementing space (Zheng et al., 2022). Each wall's thickness was measured using the cursor detail tool, and subtle modifications were made to preserve the anatomy while maintaining a minimum thickness of 2 mm for each wall. Zircona‐reinforced lithium silicate ceramic blocks (Celtra Duo, Dentsply Sirona) were used for milling with the help of CEREC MCXL milling machine using 12 s step and cylinder‐pointed burs. After every four milling a new pair of bur replaced the old one (Ceylan et al., 2022). Finally, a total number of 36 fabricated endocrowns were crystallized in furnace (CEREC speed fire, Dentsply Sirona) at 820°C.

### 2.4 Measurement of marginal and internal discrepancies

**Figure 2:**
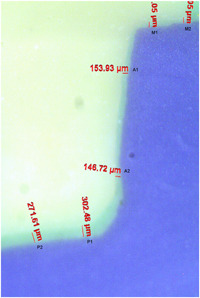
Representation of measurement positions for marginal and internal adaptation in cross‐sectional cut of replica. Green light layer represents the space between the tooth and endocrown; M1 and M2: absolute marginal discrepancy. A1 and A2: axial discrepancies. P1 and P2: pulpal discrepancies.

Marginal and internal discrepancies were measured before the cementation. One expert clinician seated each endocrown on its corresponding tooth and adjusted them up to three times to ensure seating of the restoration. The replica technique was used for the discrepancy measurements. For this reason, four sections were indicated on each specimen (buccal, lingual, mesial, and distal). After that, a green additional‐silicon‐based impression material with low viscosity (Eldosent, Pontoise, France) was applied to the bonding surfaces of endocrowns. Endocrowns were then seated on their corresponding teeth and were kept under finger pressure, which was applied in the occlusal direction until the polymerization was completed. Subsequently, the endocrown was removed while a thin layer of impression material was attached to the intaglio surface of the tooth, representing a replica of the space between the tooth and endocrown. Next, to stabilize the green layer, an additional‐silicon‐based impression material with high viscosity and in yellow color (Eldosent, Pontoise, France) was inserted into the tooth cavity and removed alongside the green layer after the set time had passed. These two layers were eventually stabilized by applying a purple layer of silicone‐based medium‐viscosity material (Eldosent, Pontoise, France) that resembled the tooth in final impression. The impressions were cut from the center in the mesio‐distal and bucco‐lingual direction by a sharp scalpel and were examined under the stereomicroscope (Olympus SZX16, Olympus) with magnification of 1.6x. Six measuring points were selected on each section and absolute marginal, axial, and pulpal discrepancies were measured (2 points for each discrepancy measurement). Microbin digital camera (Microteb, Tehran, Iran) was used for capturing the stereomicrographs at a magnification of 1 and then they were transferred to the computer. A total of 864 points (6 measuring points × 4 sections × 12 endocrowns × 3 groups) were analyzed using the axiovision microscope imaging software release 4.8 (Zeiss). All measurements were recorded in micrometers (μm) and were carried out by one expert operator (Figure 2).

### 2.5 Endocrown cementation

Before cementation, all restorations were cleaned by alcohol in an ultrasonic cleaner for 2 min. The intaglio surface of restoration was etched with 9% hydrofluoric acid gel (Ultradent Porcelain Etch) for 20 s; then rinsed thoroughly for 60 s, and dried. After that, the ceramic primer (Clearfil, Kuraray Noritake Dental Inc.) was applied to the restoration and after a few seconds, it was air‐dried. Additionally, prepared tooth surfaces were selectively etched with 37% phosphoric acid (Ultra‐Etch, Ultradent products) for 10 s, rinsed, and dried. Tooth primer (Kuraray Noritake Dental Inc.) was applied to tooth surfaces for 20 s and thinned with the help of oil‐free compressed air. Finally, the Panavia V5 cement (Kuraray Noritake Dental Inc.) was dispensed into the inner surfaces of the restoration and the endocrown was seated on its corresponding tooth. Following the correct placement of the restoration, the excess cement was partially cured for 3–5 s. The semi‐cured excess cement was then cleaned with an explorer. Finally, each surface was cured for 40 s. To ensure complete deep polymerization of the cement, the restorations were left in place untouched for 3 min, since studies have shown that light does not pass‐through CAD/CAM materials beyond 4 mm thickness (Butterhof & Ilie, 2020; El‐Askary et al., 2022).

### 2.6 Fracture resistance test

After the cementation, specimens were loaded vertically in universal test machine (Z050, Zwick Roell) until fracture occurred to measure fracture resistance. The loading piston was centered along the long axis of teeth and the load was applied with an 8 mm diameter steel ball. The thrust speed of the machine was 0.5 mm/min. Mode of failure also was recorded as tooth fracture, endocrown fracture, or both.

### 2.7 Statistical analysis

Collected data were analyzed using statistical SPSS 25 software (IBM SPSS Statistics for Windows, Version 25.0. Armonk, NY: IBM Corp). The Kolmogorov–Smirnov test was used to verify the normality of the collected data. One‐tailed one way analysis of variance (ANOVA) and post hoc analysis (Tukey's test) were used to compare fracture resistance and discrepancy of restorations of all groups. *p* < .05 was considered to be statistically significant.

## 3 RESULTS

Descriptive statistics (mean and standard deviation) for both adaptation (marginal, axial, and pulpal discrepancies) and fracture resistance of the three groups are presented in Table 1. A statistically significant difference was observed among the groups regarding the axial discrepancy (*p* = .022). Results of the post hoc analysis (Tukey's test) revealed that group A had significantly higher amounts of axial discrepancy compared to group B (*p* = .001) and group C (*p* = .003). The maximum mean axial discrepancy value was found in group A (129.06 ± 26.35 μm), while the minimum value was observed in group B (96.65 ± 19.39 μm). However, preservation of the intact axial walls did not result in a statistically significant decrease in marginal (*p* = .21) and pulpal (*p* = .31) discrepancy values.

**Table 1:** Mean and standard deviation values of discrepancies (µm), fracture resistance (N), and the modes of failures.

	Group A	Group B	Group C
**Adaptation (µm)**			
Axial discrepancy^a^	129.06 ± 26.35	96.65 ± 19.39	108.99 ± 19.33
Marginal discrepancy	170.32 ± 53.07	147.28 ± 42.60	160.99 ± 30.25
Pulpal discrepancy	216.08 ± 49.96	205.43 ± 44.19	223.46 ± 41.38
Fracture resistance (*N*)	4154.08 ± 1931.424	2526.83 ± 533.037	3109.92 ± 1392.367
**Modes of failure (number of specimens)**			
Tooth fracture	0	0	0
Endocrown fracture	4	6	4
Both	8	6	8

*Note*: Group A: no complete wall; Group B: buccal wall remained; Group C: buccal and mesial wall remained.

^a^ Statistically significant at *p* < .05.

One‐tailed one way ANOVA showed no statistically significant increase in fracture resistance due to preservation of intact axial walls (*p* = .51). Also, Post hoc Tukey's test revealed that there was no significant difference between groups.

The fracture mode is also presented in Table 1. The fracture of both tooth and endocrown was predominant in groups A and C (66%). Group B demonstrated an equal percentage of endocrown fracture and tooth/endocrown fracture (50%). Tooth fracture alone was not observed in any of the groups.

## 4 DISCUSSION

This in vitro study aimed to assess the effect of the preparation design on the adaptation and the fracture resistance. Based on the results, the hypothesis that keeping the intact axial walls could improve the adaptation and the fracture resistance of the endocrowns was partially accepted. While keeping at least one of the axial walls could improve the adaptation only in the axial area it did not change the fracture resistance. Several factors have been discussed in the previous studies that can play a role in the adaptation of the endocrowns. These factors include the material type (El Ghoul et al., 2020; Kassem et al., 2020; Saglam et al., 2020; Zimmermann, Valcanaia et al. 2019), the measurement method (Boitelle et al., 2014), the restoration depth (Gaintantzopoulou & El‐Damanhoury, 2016; Rocca et al., 2018; Shin et al., 2017), and the fabrication method (Yun et al., 2017). To the best of the authors' knowledge, no study has evaluated the effect of the preservation of the axial walls on the marginal and internal discrepancies of the endocrowns.

To achieve a reliable evaluation of the adaptation, sample standardization was done by selection of the human natural teeth with approximate similar dimension, using the same rotary system for all the root canal treatments, considering the same cement spacer (60 μm) and at least 2 mm occlusal thickness for all the specimens and sectioning all the replicas in the same position for a perpendicular view on the stereomicroscope.

The measuring technique seems to be one of the most influential parameters because of its direct impact on the results of the study (Trifkovic et al., 2012). Several 2‐dimensional (2D) and 3‐dimensional (3D) methods have been used for measuring the marginal discrepancies including direct visualization (Abduo et al., 2010), dental explorer (Abduo et al., 2010), cross‐sectional technique after cementation (Son et al., 2019), impression technique (the replica technique) (Taha et al., 2018a), triple scan method (TSM) (M. Hasanzade et al., 2020), and microcomputed tomography (μCT) (Shin et al., 2017). Among all of these methods, the replica technique is one of the most commonly used ones (Falk et al., 2015; Son et al., 2019). Although studies have demonstrated that 3D techniques can provide more accurate measurements than replicas, these techniques are not applicable to clinical situations and can only be employed in lab studies (Boitelle, Tapie, Mawussi, & Fromentin, 2018; Hasanzade, Koulivand, Moslemian, & Alikhasi, 2020). Simplicity, non‐invasiveness, repeatability, and affordability are among other reasons that have made this 2D method so useful both in in‐vitro and in‐vivo studies (Praça et al., 2018; Son et al., 2019). One of the disadvantages of this method is the possibility of dimensional change and tearing of the impression material (Park et al., 2017). In this study, additional‐silicon‐based impression material was used that has an excellent dimensional stability (Lozano et al., 2021).

Another important factor to consider when it comes to measuring the internal and marginal discrepancy, is the type of the intra‐oral scanner. Digital dentistry requires scanning accuracy to reproduce the prepared tooth surface. Higher accuracy and precision can result in higher compatibility and better internal and marginal adaptation of the restoration, which ultimately increases success rate (Ferrini et al., 2019; Hayama et al., 2018).

In this study, adaptation of endocrowns was assessed by measuring the internal and absolute marginal discrepancy. For better comparison, the internal measurements were divided into two different areas: axial and pulpal. According to the results of this study, teeth with the preserved axial walls showed better adaptation only in the axial region. Although no specific cut‐off point has been suggested regarding the internal discrepancy, discrepancies between 50 and 200 μm have been reported in the studies (Arcuri et al., 2019; Nawafleh et al., 2013). The mean axial discrepancy of the tested groups was in the range of 96.65–223.46 μm. Group A showed the largest axial discrepancy (129.06 ± 26.35 μm), followed by group C (108.99 ± 19.33 μm) and group B (96.65 ± 19.39 μm) which showed the smallest axial discrepancy. The mean pulpal discrepancy of the tested groups was in the range of 205.43–223.46 μm which is higher than the clinical acceptable range. The high amounts of the pulpal discrepancy which is consistent with the previous studies (El Ghoul et al., 2020) can have two main reasons: the limited optical depth of the scanner and the overmilling of the pulpal floor (Hasanzade et al., 2019; Zimmermann et al., 2019). The clinically acceptable range for the marginal discrepancy has been reported to be between 75 and 160 μm (Boitelle et al., 2014; Nawafleh et al., 2013). The mean values for the marginal discrepancy of all the groups in this study were nearly in accordance with this range (159.46 ± 42.75). This amount was higher than the one which Taha et al. (2018a, 2018b) reported in measuring the marginal adaptation of the Celtra Duo endocrowns (45.8 ± 16.16). One of the possible reasons could be that they measured the vertical marginal discrepancy which is clearly lower than the absolute marginal discrepancy (Holmes et al., 1989). In another similar study, Zimmermann et al. (2019) measured the marginal discrepancy of the endocrowns fabricated from Celtra Duo, which is slightly higher than the values in this study (182.3 ± 24.0 μm). This could be due to the differences in the measuring methods as they used the 3D digital measurement technique. In a recent study, Soliman et al. (2022) reported a lower amount of marginal discrepancy for Celtra Duo endocrowns (29.54 ± 6.32 μm). They measured the vertical marginal discrepancy and used a more advanced intra‐oral scanner (Primescan), which is reported to have more precision and trueness than Omnicam. Possibly this explains why this study's reported amounts were lower (Gurpinar & Tak, 2022; Soliman et al., 2022).

Fracture resistance is one of the main factors for evaluating the long‐term success of the CAD/CAM fabricated restorations (Elsaka & Elnaghy, 2016). The structural destructions that accumulate during mastication can lead to the restoration's fracture (Chang et al., 2009). The results of this study showed that keeping the intact axial walls did not have any effect on the improvement of the fracture resistance. This result could be attributed to the presence of the surrounding enamel in all the groups that can contribute to the same stress distribution (Tribst et al., 2018). In a similar study, Taha et al. (2018b) evaluated the fracture resistance of endocrowns in the teeth with axial reduction and concluded that the teeth with axial reduction were not different from the ones without axial reduction in terms of the fracture resistance.

The mean fracture resistance values of all of the three groups were between 3109.9 and 4154 which is higher than the similar articles (Dartora et al., 2021; Sedrez‐Porto et al., 2016). The main reason for the higher values of the fracture resistance in this study could be related to not subjecting the specimens to thermocycling which can alter the results of the static loading. A noticeable finding was that in this study, the values recorded for all the groups were above 850 N, which is the clinically acceptable threshold in the molar area according to Waltimo and Könönen (Waltimo & Könönen, 1993). Also comparing the modes of failure showed that group B had the most endocrown fracture. This is explained by the fact that the presence of the buccal wall can result in a thinner restoration in that site and therefore more stress concentration and endocrown fracture (Tribst et al., 2018).

One of the limitations of this study was that it was an in‐vitro study, which may not resemble the exact clinical situation. Another limitation of this study was the high standard deviation in fracture resistance results. Despite our efforts to reduce heterogeneity within the variables of groups, the standard deviation was still high. Moreover, regarding the fracture resistance experiment, it would have been better to analyze the samples to determine whether they were repairable. Thus, in vivo studies evaluating the mode of fracture in relation to their repairability are recommended to determine the effect of axial wall preservation on endocrown features.

## 5 CONCLUSION

Within the limitations of this study, it was concluded that keeping at least one of the axial walls of the teeth at its original height during the endocrown preparation can reduce the amount of the axial discrepancy and improves adaptation of the restoration, though it did not affect fracture resistance.

## AUTHOR CONTRIBUTIONS

**Hamid Jalali and Marzieh Alikhasi**: have made substantial contributions to conception and design of the study. **Samar Jalali and Ali Abdolrahmani**: have been involved in acquisition of data. **Mohamad javad Kharazi fard and Ali Abdolrahmani**: made contributions in analysis and interpretation of data. **Samar Jalali**: has been involved in drafting the manuscript or revising it critically for important intellectual content. All authors of this paper have given final approval of the version to be published. **Hamid Jalali**: has agreed to be accountable for all aspects of the work in ensuring that questions related to the accuracy or integrity of any part of the work are appropriately investigated and resolved.

## CONFLICT OF INTEREST STATEMENT

The authors declare no conflicts of interest.

## Data Availability

The data that support the findings of this study are available from the corresponding author upon reasonable request.
